# The New York State Medical Society, and Medical Fees

**Published:** 1857-09

**Authors:** 


### EDITORIAL DEPARTMENT.

*The New York State Medical Society, and Medical Fees.*—The late decision of Judge Marvin, of the Supreme Court, in the case of Dr. Hill, against the Erie County Medical Society, is one affecting the interest of the profession of the entire State, although originating in action had by a local society. It has been the interest of those who have prayed for the defeat of our county society, to represent that the action was purely sectional, instigated for the purpose of the attainment of personal ends, and—that it was very doubtful if the subject were brought to the notice of the State Society, whether it would sustain the course of the County Society.

The recently published volume of the Transactions of the State Medical Society, at its annual session, held at Albany in February last, exhibits the fact, that the State Society has not deemed it beneath its notice or dignity, to speak out upon the subject of the inadequacy of the compensation paid for services rendered to the public. We propose to copy from the Transactions, all that refers to this subject.

From the inaugural address of the President, Dr. Alden March, delivered on the first day of the session, we make the following extracts:

"It is also thought proper to call the attention of the society to the consideration of another subject—one in which some of my constituents, and I doubt not many of the members of this honorable body, feel a deep interest. I refer to the services physicians and surgeons are occasionally called upon to render to the county, under the direction of coroners, in making post-mortem examinations by dissection, in cases where murder is suspected. I need not advert to the necessity of having a properly qualified medical man to make such examinations—to the time spent—to the risk of life and limb; and to the responsible and vexatious position in which the witness is often placed before a court of justice. We do not wish to give the public the impression that our profession is mercenary — that we are too much influenced by pecuniary reward. You, as well as the whole world know, that physicians and surgeons are more heavily taxed in taking care of the poor, than any other profession, trade, or calling.

"Most certainly 'the laborer is worthy of his hire.' The Medical Society of the County of Albany, not long since, resolved to be no longer imposed upon, or insulted by the supervisors, by having a reasonable charge for post-mortem dissections, cut down to the pay of an ordinary day laborer. The following is the rate of charges for such services, agreed upon at a special meeting of our society, held in December last;

"For making post mortem examinations with dissections in criminal cases, where person is committed and tried, from $15 to $50.

"For making post-mortem examinations with dissection, to ascertain cause of death when crime is suspected, $10.

"For examining bodies and testifying as to cause of death, without dissection, $5.

"For making examination and giving certificate under oath in cases of insanity, $3.

"I find that this subject, as well as those that relate to professional services and attendance on the inmates of alms-houses, jails, work-houses, &c., was brought before this Society in 1850, by Prof. James P. White, of Buffalo. On that occasion it appears that the following resolutions were unanimously adopted:

"1st. '*Resolved*, That this Society recommend the several County Societies to arrange a minimum tariff of prices for those several offices and services within their limits, which, whilst it shall afford a reasonable compensation for the attention bestowed, shall at the same time be so much only as will meet the approbation of the more reflecting members of community, and so low as to afford no reasonable excuse for its infraction.

"2d. '*Resolved*, That it shall be considered derogatory to the character of any member of the profession to violate any such bill or tariff of prices by underbidding when it shall have been regularly adopted by the Society of the county in which he may reside.'

"This subject has been thought of sufficient importance, and of a character so general, as to demand the attention of the Society. But whether by any definite action or otherwise, will be for the members to determine."

Immediately succeeding the address, on the motion of Dr. Saunders, of Schuyler County, the following was adopted:

"*Resolved*, That a committee of five be appointed to take into consideration so much of the President's opening address as relates to post-mortem examinations before coroners, and the charges for the same, and that they report at this meeting."

We judge that the committee referred to in the resolution, was not appointed, as we do not find their names in the minutes of the proceedings; but in the transactions of the next day's afternoon session, we read as follows:

"Dr. Crandall presented the following, which was adopted:

"*Whereas*, It is a well-known fact that in the prosecution of criminal justice, members of the medical profession are often required to leave their homes and their business and attend for days, and sometimes even weeks, upon the courts, to give their testimony in cases of which they have no personal knowledge, being called solely as medical men to give professional opinions and conclusions founded upon the evidence which may be adduced in the legal examination. In this way the physician is often forced away from his legitimate business to his own disparagement, and not unfrequently to the serious neglect of the sick depending upon his daily ministrations. We consider this expensive and unrequited labor a serious and oppressive tax upon the medical profession, and one which is occurring so frequently that it calls loudly for legislative address; therefore,

"*Resolved*, That the practice of medicine is attended with peculiar toil, self-sacrifice and responsibility, and that while the medical profession acknowledges its obligation to meet all the just demands upon it of the community, it would also claim a reciprocal obligation to be relieved of all unreasonable and unnecessary burdens.

"*Resolved*, That the professional skill and knowledge of the physician is his own, having been purchased at the expense of many anxious and toilsome hours, and the liberal outlay of his means; and if it be worth anything to others, it should also be valuable to himself, and the public have no more right to claim its gratuitous employment than that of other professional men; and if the member of the bar is permitted to make a public charge for his services in prosecuting or advocating a criminal case, the physician who is called upon to give professional opinions and the results of professional experience in the same case, aught also to claim redress in the same way.

"*Resolved*, That physicians throughout the State and the various medical societies, should unite in petitions to the Legislature for such an enactment as shall provide a sufficient remuneration for the services and knowledge of the physician when claimed by the courts in the prosecution of criminal justice."

In the proceedings of Thursday morning, the third day of the session, we find the following recorded:

"Dr. Hall presented the following, which was adopted:

"*Resolved*, That this society recommend to the county societies of this State to adjust a scale of fees for all medico-legal services. Also that the Albany County Society's scale be published in the Transactions, as a future guide for others." N.

